# Multifactorial Benchmarking of Longitudinal Player Performance in the Australian Football League

**DOI:** 10.3389/fpsyg.2019.01283

**Published:** 2019-05-31

**Authors:** Sam McIntosh, Stephanie Kovalchik, Sam Robertson

**Affiliations:** ^1^Institute for Health & Sport, Victoria University, Melbourne, VIC, Australia; ^2^Western Bulldogs Football Club, Melbourne, VIC, Australia

**Keywords:** decision support, performance analysis, data visualisation, player evaluation, team sport

## Abstract

This study aimed to develop a model to objectively benchmark professional Australian Rules football (AF) player performance based on age, experience, positional role and both draft type and round in the Australian Football League (AFL). The secondary aims were to identify the stage of peak performance and specific breakpoints in AF player performance longitudinally. AFL Player Ratings data were obtained for all players (*n* = 1052) from the 1034 matches played during the 2013–2017 seasons, along with data pertaining to the abovementioned player characteristics. Two separate linear mixed models revealed that all factors influenced player performance, with age and experience the strongest in each model, respectively. *Post hoc* Tukey tests indicated that performance was affected by age at each level up until the age of 21 (effect ranging from 0.98 to 3.70 rating points), and by experience at the levels 1–20 and 21–40 matches in comparison to all higher levels of experience (effect ranging from 1.01 to 3.77 rating points). Two segmented models indicated that a point of marginal gains exists within longitudinal performance progression between the age levels 22 and 23, and the experience levels 41–60 and 61–80 matches. Professional sporting organisations may apply the methods provided here to support decisions regarding player recruitment and development.

## Introduction

Identifying when peak performance typically occurs in athletes is an important consideration within professional team sport organisations. Specifically, at what point in an athletes career are they likely to reach their peak. Such information can be used to inform contracting as well as the make-up of team rosters. The identification of peak performance can be measured longitudinally on various time series including the age of an athlete, amount of years within a professional program and their match’s experience (Torgler and Schmidt, 2007). Additionally, various type of peaks have been investigated within the notational team sport literature, including when an athlete is at their physiological peak (Reilly et al., 2000), when they reach their peak market value (Kalén et al., 2019), as well as when their on-field performance is at its peak (Fair, 2008; Bradbury, 2009; Dendir, 2016). Although peak performance has been well documented longitudinally for age in individual sporting events (Schulz and Curnow, 1988; Allen and Hopkins, 2015; Longo et al., 2016), its identification within team sports may be more complex. This complexity primarily arises due to the difficulty objectively outlining individual performances given that there are no quantifiable outcomes which occur directly from player actions in most team sports (Travassos et al., 2013; Robertson et al., 2015). Additionally, there is an increased importance of specific skill demands required in team based sports, including non-physical abilities such as experience and strategic knowledge (Bradbury, 2009), as well as the complexity of accounting for differences individual playing roles.

Despite this, individualised assessment of match performance in professional team sports is commonplace. This includes both subjective assessments of performance, as made by team coaches, management and within the media, as well as objective assessments made through data-driven techniques (Carling et al., 2008; Bonney et al., 2019). Although subjective assessments are often made by those in influential decision making positions (i.e., coaches), there has been a change within professional sport organisations toward supporting decisions with objective assessments (Maymin, 2017). Concurrently, there has been an increasing amount of data-driven techniques proposed in literature regarding assessing individual player performance in team sport on a quantitative scale. Some examples include Radovanović et al. (2013) who developed a player efficiency rating, which objectively measures a player’s productivity in basketball based on player actions such as points, assists, rebounds, steals and turnovers, and their outcomes. Similarly, McHale et al. (2012) developed a player performance index to rate the performance of players in the top two leagues of English soccer on a quantitative scale including items such as match contributions, winning performance, match appearances, goals scored, assists, and clean sheets.

Australian Rules football (AF) is a dynamic invasion team sport played between two opposing teams consisting of 22 players each (18 on the field and four interchange). In the elite competition of AF, the Australian Football League (AFL), players can be drafted to a professional club and begin playing as early as the age of 18, with various players managing to continue playing into their middle-to-late thirties. There has been a substantial amount of research developed in AF to identify the physical and technical characteristics of individual players with respect to match performance (Young et al., 2005; Veale et al., 2008; Mooney et al., 2011; Tangalos et al., 2015; Woods et al., 2016). However, to our knowledge there has been no research examining longitudinal player performance in professional AF. However, various studies exist in the wider notational sport literature which investigate longitudinal player performance, predominantly on identifying the age at which peak performance occurs. Examples include Dendir (2016), who used mixed effects models, and identified that the peak age of performance in the top four professional soccer leagues varied between 25 and 27, depending on position. Kalén et al. (2019) similarly looked to identify the peak age of performance in professional soccer. Using a one-way ANOVA and linear regression they found that a significant longitudinal shift in peak age has occurred from 24.9 years in 1992–1993 to 26.5 years in 2007–2018. Using a random effects model Bradbury (2009) investigated peak performance of skills in baseball, finding that overall performance peaks around the age of 29. Specifically, athletic skills such as hitting and running peak earlier, whilst skills based on experience and knowledge such as drawing walks, peak later. Fair (2008) also examined the estimated age effects in baseball. Using a non-linear fixed effects regression, they found that the peak age and begin of decline in performance occurred around the age of 26 years for pitchers, and 28 years for batters.

In the abovementioned studies, both Dendir (2016) and Fair (2008) emphasise that considerations or assumptions must be made about other factors when assessing longitudinal player performance. Notably, a player’s position and their level of experience. In addition to these factors, another consideration is the position at which players are selected in their respective draft. Studies such as O’Shaughnessy (2010) have looked to develop a valuation system for the AFL National Draft, indicating that earlier selections are valued more highly on the basis that clubs can select the best available player in the pool.

In addition to identifying peak player performance, longitudinal research has also looked to identify whether specific changes in trends occur within a time series. Within sport performance, this research has consisted of identifying longitudinal changes in trends of physical performance (Fransen et al., 2017; Towlson et al., 2018), game related statistics (Lorenzo et al., 2019), and gameplay (Wolfson et al., 2015; Woods et al., 2017), as well as whether external factors such as a player’s contract status effect performance (Gómez et al., 2019). Though this type of model has not been applied to player performance in team sports, the use of this procedure would allow for the construction of a model to identify whether a breakpoint in longitudinal player performance exists.

The ability to benchmark player performance longitudinally is inherently valuable to many sports, and could be used to support organisational decisions regarding player contracting, recruitment and development (Kalén et al., 2019). In the AFL, there is a large emphasis on decisions relating to player contracting and recruitment as clubs are confined in their ability to remunerate players by a salary cap. Decisions relating to player development are also vital, as clubs do not have the opportunity to attain additional players within season. As such, the ability to inform these decisions based on comparisons of player performance against model-expected performance, or the ability to forecast future performance is advantageous. Further, a greater understanding of when performance progression is at its maximum, or conversely when progression is expected to deteriorate, could have important implications for the type of skill development implemented for specific individuals.

There are various player performance measures which are produced commercially within the AFL. The “AFL Player Rankings” is produced by statistics provider Champion Data Pty Ltd., measures player performance by awarding players a fixed value for specific performance actions. The values for these actions were determined relative to their observed relationship to team winning margin (Herald Sun, 2016). Alternatively, the “AFL Player Ratings”, which is also produced by statistics provider Champion Data Pty Ltd., measures player performance based on the principle of field equity. In this metric, points are awarded to (or deducted from) a player based on contextual information relating to each possession, relative to how much their actions increase or decrease their team’s expected value of scoring next (Jackson, 2009; McIntosh et al., 2018).

The primary aim of this study was to develop a model to objectively benchmark AFL player performance whilst considering their age, experience, positional role and both draft type and round in which they were selected. The secondary aims were to identify the stage of peak performance and specific breakpoints in player performance longitudinally. To achieve these, this study will consider the player characteristics and model types outlined in the abovementioned literature.

## Materials and Methods

### Data

The AFL Player Ratings were utilised as the objective measure of player performance in this study due to its validity and its equity-based nature (Jackson, 2009; McIntosh et al., 2018). In this metric, a player’s overall match performance is measured by the overall change in equity that is created by that player’s actions during the course of a match (Jackson, 2009). The change in equity is determined by expected value of their team scoring next. These expected values are based on contextual information relating to possessions (i.e., field position, pressure from opponents, possession outcome) collected from all AFL matches preceding back to the 2004 season (Jackson, 2009).

These AFL Player Ratings were obtained from Champion Data Pty Ltd. for all 1034 matches played throughout the 2013–2017 AFL seasons. This included 22 matches played by each team during the regular season rounds, as well as a total of nine matches played throughout the finals series each season. One match was abandoned prior to play during the 2015 season. The AFL Player Ratings data were expressed as a mean season rating for each player across each of the five seasons. The sample included a mean of 3.15 seasons per player (±1.55 SD) among 1052 unique players, giving a total sample size of *n* = 3317.

Data pertaining to player characteristics were also collected in order to assess their relationship with performance. Age (determined by the players age at 31st December of the previous year), experience (determined by the number of AFL matches played, independent of seasons, and taken at the conclusion of each season), positional role classification (determined by Champion Data’s classification at the conclusion of each season; classifications outlined in Appendix Table A1) and the characteristics of the draft (draft types outlined in Appendix Table A2) in which each player was first selected by an AFL club were all collected as descriptive variables. Prior to data collection, the study was approved by the relevant human research ethics committee.

### Data Analysis

For modelling purposes, various aspects of the data required transformation. All characteristics were considered as categorical variables. Categorisation levels for age and experience were determined by evaluating the change in Akaike’s Information Criterion for differing amounts of categories (Akaike, 1987). Sixteen categories for both characteristics were chosen by identifying the minimum number of categories at which the point gains in Akaike’s Information Criterion became minimal (<10). This allowed for discretisation that balanced model fit and complexity (Bozdogan, 1987). Age was expressed as integer categories (18, 19, 20, …, 33+), where due to the limited sample size of players aged 33–40 years, data were combined into one category. Experience was expressed in intervals of 20 matches (1–20, 21–40, 41–60, …, 301+), where all players with 301 or more matches experience were similarly combined into one category due to the limited sample size. Categorisation levels for draft selection were arbitrarily expressed over ten levels relative to the type and round in which they were first selected by an AFL club (five levels for National Draft rounds 1 to 5+, four levels for Rookie Draft rounds 1 to 4+, and one category for the Preseason Draft). Due to the limited sample size of players drafted after round five of the national draft, after round four of the rookie draft, and in total from the preseason draft, data were combined into one category for each draft type. Positional role classification was expressed across the seven levels as determined by Champion Data (general defender, key defender, general forward, key forward, midfielder, midfield-forward, and ruck).

Further, as part of the entry concessions given to newly established clubs, the Gold Coast Suns and the Greater Western Sydney Giants, 45 players from the dataset were drafted to AFL clubs prior to the 2011, 2012, and 2013 AFL seasons via non-traditional draft methods. Considering the circumstances of these concessions, all players drafted via methods of zone selection, as an underage recruit, through the AFL mini-draft, as an AFL initiative or were pre-listed by an AFL club (*n* = 42), were considered as first round selections within the national draft. Further, those drafted after being overlooked in the prior year’s national draft (*n* = 3) were considered as first round selections within the rookie draft.

### Statistical Analysis

Descriptive statistics for age and experience, and how they relate to AFL Player Ratings [mean ± 95% confidence intervals (CI)] were obtained. The number of matches played per season and proportion of players were also collected and plotted across age and experience. Prior to undertaking the main analyses, Spearman’s correlation analyses were employed to determine the extent of collinearity between each of the four player characteristics. This analysis was undertaken using the *Hmisc* package (Harrell, 2017) in the R statistical computing software version 3.3.2. (R Core Team, 2016). This analysis revealed a strong association between age and experience (*r* = 0.83), whilst all remaining associations were weak (*r* < 0.15). As a result, separate models were created throughout the further analyses, utilising age and experience as the independent variables in each.

To determine the extent to which these characteristics affect performance, linear mixed models were applied using the *lme4* package (Bates et al., 2015). Two separate models were created, each incorporating either age or experience, with all other factors included in both. This particular approach was used to control the variability created by the repeated measures data on each player. Specifically, the factors of interest (age, experience, positional role, and draft selection) were treated as fixed effects, and player as a random effect in both models. Each model took the form of:

$${\text{PR}}_{\text{ps}}=\beta_{0}+\beta_{1}{\text{X}}_{\text{ps}}+\beta_{2}{\text{Y}}_{\text{ps}}+\beta_{3}{\text{Z}}_{\text{p}}+\alpha_{\text{p}}+\varepsilon_{\text{ps}}$$

where PR_ps_ is the AFL Player Rating average of player *p* in season *s* (s = 2013–2017). β_0_, β_1_, β_2_, and β_3_ are fixed coefficients, and *X*, *Y*, and *Z* are observed covariates. In model (1), *X*_ps_ and *Y*_ps_ represent the player’s age and positional role for the corresponding season, respectively, whilst *Z*_p_ represents the category outlining the player’s draft selection, which stays consistent between seasons. The parameter ∝_p_ is a player random effect, which makes the intercept of the model specific to each player and allows for individualised performance projections. The player random effect is treated as constant across seasons and each effect is a draw from a normal distribution with equal variance for all players. The parameter 𝜀_ps_ denotes the player-season residual error. Model (2) takes the exact same form as model (1), however, *X*_ps_ instead represents a player’s experience for the corresponding season.

Based on the fixed effects estimates, benchmark levels of performance were plotted (∝_p_ = 0) for age and experience, respectively, where means and 90% prediction intervals (PI) are averaged over the levels of positional role and draft for both. A *post hoc* Tukey test was performed to adjust for multiple comparisons, and to determine whether performance was different within each level of age and experience, and thus identifying a hypothesised breakpoint in performance. To further assess whether a breakpoint exists in each of the linear mixed models, a segmented model (or “piecewise linear model”) was fit to the data to estimate if a change in the trend of the data occurs. This analysis was undertaken using the *segmented* package (Muggeo, 2008). As a result of the *post hoc* Tukey tests, we specified the levels 22 for age, and 41–60 for experience as the hypothesised break points. Within this analysis, these points are used as starting points for which the model uses to estimate breakpoints. A level of significance was accepted at *p* < 0.01 in all analyses.

## Results

**FIGURE 1:**
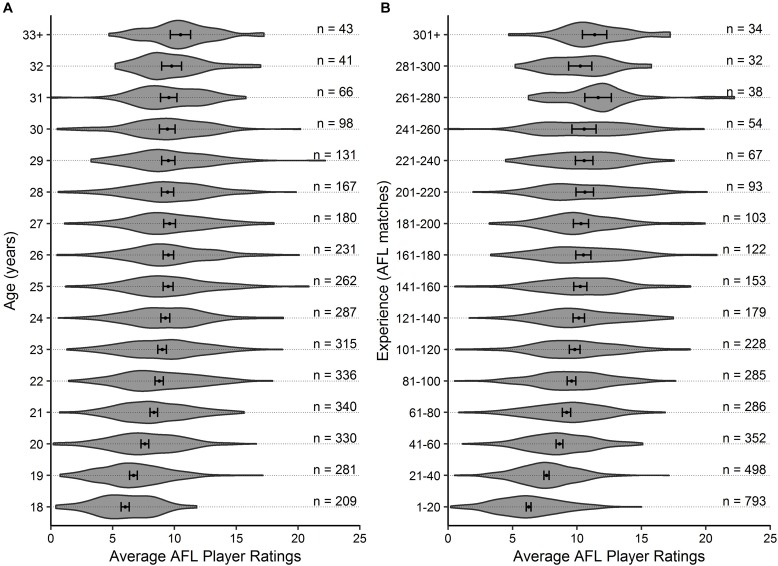
Violin plot outlining the density of the average AFL Player Ratings (±95% CI) for **(A)** age and **(B)** experience, respectively. The number of observations in each group are outlined.

Descriptive statistics are outlined in Figure 1, 2 for age and experience, and positional role and draft, respectively. Figure 3A highlights that the proportion of players competing in the AFL is at its highest at ages 20–22, and then declines with each consecutive age level thereafter. Further, Figure 3B highlights that the proportion of players is highest in the least experienced group (20 matches or less), and similarly declines with each consecutive category level of experience thereafter. On the contrary, Figure 4 indicates that the average number of matches played per season increases with both age and experience.

**FIGURE 2:**
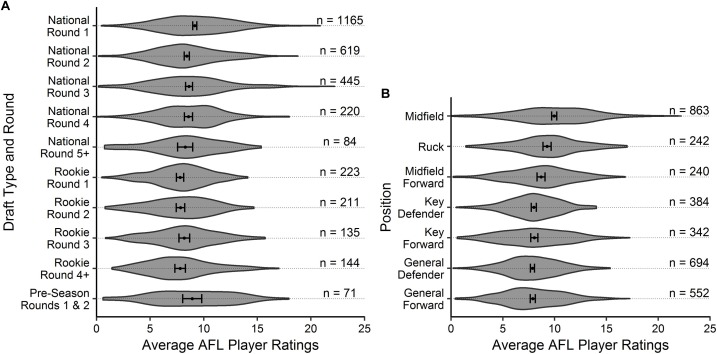
Violin plot outlining the density of the average AFL Player Ratings (±95% CI) for **(A)** draft and **(B)** positional role, respectively. The number of observations in each group are outlined.

**FIGURE 3:**
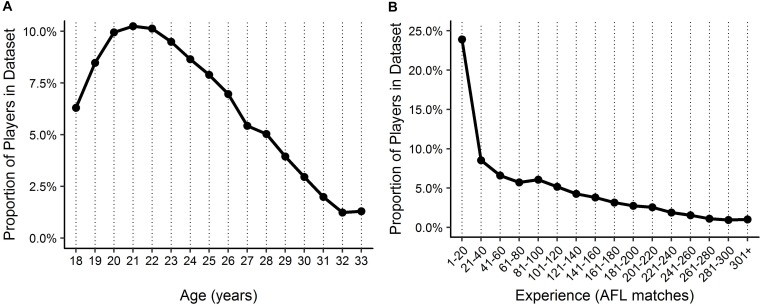
Proportion of players in the dataset by **(A)** age and **(B)** experience.

**FIGURE 4:**
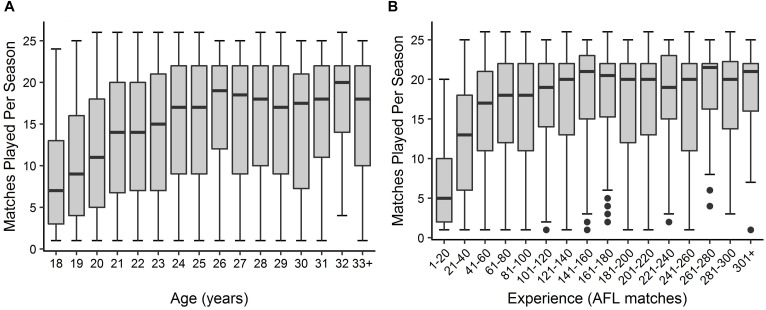
Boxplot outlining the distribution of matches played per season by players in each level of **(A)** age and **(B)** experience.

Results of the linear mixed models revealed that all factors affected levels of performance in both models at *p* < 0.01. Model (1) produced a root mean square error of 1.77 and Chi-square values of 356.9 for age, 98.7 for positional role and 57.1 for draft. Comparatively, model (2) produced a root mean square error of 1.82 rating points and Chi-square values of 523.5 for experience, 100.4 for positional role and 21.7 for draft. The values indicate that age and experience had the largest influence on performance in each of the models, respectively, followed by positional role. Tables 1, 2 outline the fixed effect coefficients (β_0_, β_1_, β_2_, and β_3_) for each factor level of the characteristics in each of the respective models.

**Table 1:** Model (1) fixed effect regression coefficients outlining the estimated difference in rating points from the reference level of each factor.

	Regression coefficients (±SE)
(Intercept)	7.11 (0.23)
Age 19	0.98 (0.20)
Age 20	1.93 (0.21)
Age 21	2.62 (0.21)
Age 22	3.06 (0.22)
Age 23	3.32 (0.22)
Age 24	3.39 (0.23)
Age 25	3.69 (0.24)
Age 26	3.70 (0.25)
Age 27	3.68 (0.26)
Age 28	3.31 (0.27)
Age 29	3.18 (0.29)
Age 30	2.80 (0.32)
Age 31	2.48 (0.37)
Age 32	2.56 (0.44)
Age 33+	2.46 (0.47)
Positional role Gen Def	-1.25 (0.17)
Positional role Gen Fwd	-1.13 (0.17)
Positional role Key Def	-1.128 (0.23)
Positional role Key Fwd	-1.79 (0.23)
Positional role Mid Fwd	-0.79 (0.19)
Positional role Ruck	-0.38 (0.29)
Draft National 2	-0.78 (0.23)
Draft National 3	-0.74 (0.25)
Draft National 4	-0.94 (0.32)
Draft National 5+	-1.21 (0.47)
Draft Rookie 1	-1.47 (0.32)
Draft Rookie 2	-1.62 (0.33)
Draft Rookie 3	-1.56 (0.39)
Draft Rookie 4 +	-1.75 (0.38)
Draft Preseason	-1.03 (0.57)

*Reference level for each factor were: age 18, positional role midfield, Draft National 1.*

**Table 2:** Model (2) fixed effect regression coefficients, outlining the estimated difference in rating points from the reference level of each factor.

	Regression coefficients (±SE)
(Intercept)	7.43 (0.18)
Experience 21–40	1.31 (0.14)
Experience 41–60	2.32 (0.16)
Experience 61–80	2.79 (0.18)
Experience 81–100	3.19 (0.18)
Experience 101–120	3.38 (0.20)
Experience 121–140	3.48 (0.22)
Experience 141–160	3.39 (0.23)
Experience 161–180	3.77 (0.25)
Experience 181–200	3.43 (0.27)
Experience 201–220	3.53 (0.29)
Experience 221–240	3.32 (0.33)
Experience 241–260	3.02 (0.36
Experience 261–280	3.74 (0.43)
Experience 281–300	2.46 (0.47)
Experience 301+	3.02 (0.52)
Position Gen Def	-1.17 (0.16)
Position Gen Fwd	-1.24 (0.16)
Position Key Def	-1.07 (0.21)
Position Key Fwd	-1.49 (0.22)
Position Mid Fwd	-0.74 (0.19)
Position Ruck	-0.12 (0.26)
Draft National 2	-0.54 (0.20)
Draft National 3	-0.30 (0.23)
Draft National 4	-0.27 (0.29)
Draft National 5+	-0.75 (0.42)
Draft Rookie 1	-0.89 (0.29)
Draft Rookie 2	-0.85 (0.30)
Draft Rookie 3	-0.46 (0.35)
Draft Rookie 4 +	-0.71 (0.34)
Draft Preseason	-0.49 (0.51)

*Reference level for each factor were: experience 1–20, positional role midfield, Draft National 1.*

Results of the *post hoc* Tukey test indicated that performance was affected by age at various age levels up until the age of 21 (mean differences ranged from 0.98 to 3.70 player rating points). However, no two levels above the age of 21 were seen to exhibit different levels of performance. For experience, differences were seen at the levels of 1–20 matches and 21–40 matches in comparison to all higher levels of experience (mean differences ranged from 1.01 to 3.77 player rating points), and for various experience levels in comparison to 41–60 matches. No differences were seen between any levels above this for experience.

**FIGURE 5:**
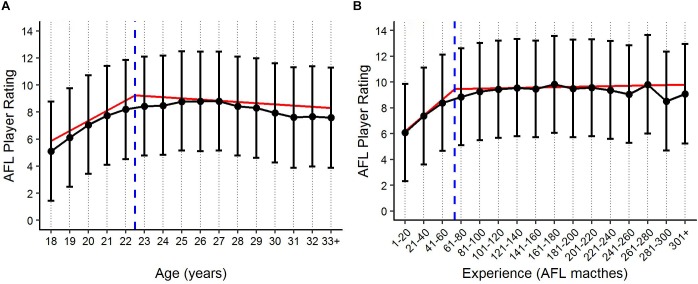
Benchmark levels of AFL Player Ratings (±90% PI) by **(A)** age and **(B)** experience, based on the fixed effects estimates. Blue *x*-axis intercept lines represent the level at which the breakpoint in performance occurs for both age and experience, respectively. Red regression lines represent the multiple linear fits of the segmented models.

The segmented models identified a breakpoint in performance for both age and experience. The results indicate that a breakpoint in age occurs between the age levels 22 and 23, where performance is seen to increase linearly 0.75 rating points per age level prior to this breakpoint, and decline linearly 0.09 rating points per age level thereafter. The breakpoint identified for experience occurs between the levels 41–60 and 61–80, where performance is seen to increase linearly 1.24 rating points per level of experience prior to this breakpoint, and then continue to increase linearly 0.04 rating points per experience level thereafter. Figure 5 displays the benchmark levels of performance for both age and experience, where player specific random effects (PSRE) are removed. *X*-axis intercept lines and regression lines were added to Figure 5 to represent the level at which the identified breakpoint in performance occurs, and the change in the trend of player performance, respectively, for both age and experience.

**FIGURE 6:**
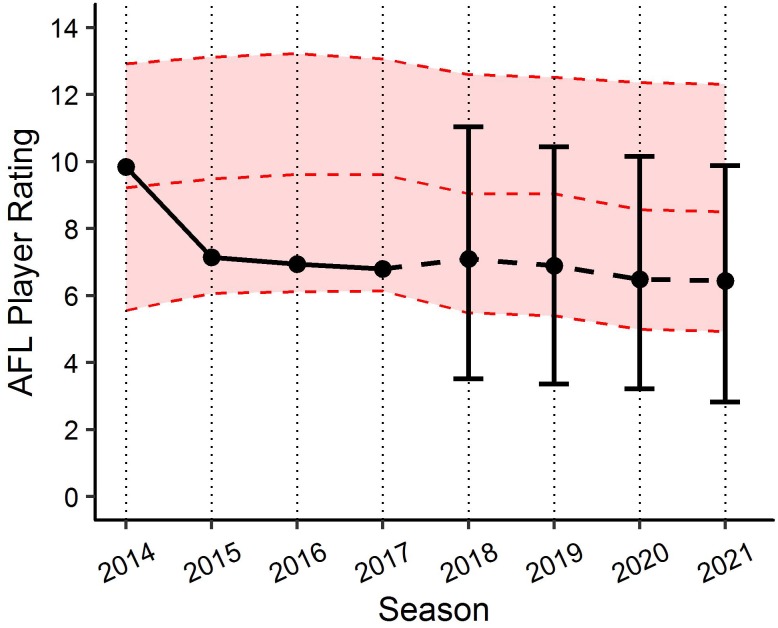
Benchmark levels of AFL Player Ratings for a specific player using the age linear mixed model. Black lines represents actual performance to 2017 and player specific expectation (±90% PI) of performance from 2018. Red ribbon represents fixed effects estimates based on characteristics of same player.

**FIGURE 7:**
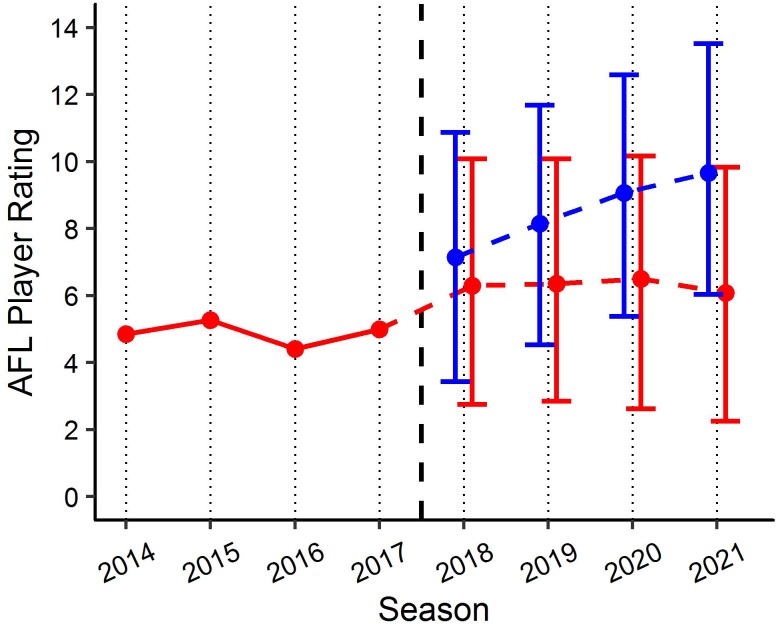
Benchmark levels of AFL Player Ratings for two specific players using the age linear mixed model. Red line represents actual performance prior to 2017. Red and blue lines indicate player specific expectations (±90% PI) of performance from 2018 for each player. Black *x*-axis intercept line indicates point of comparison.

**FIGURE 8:**
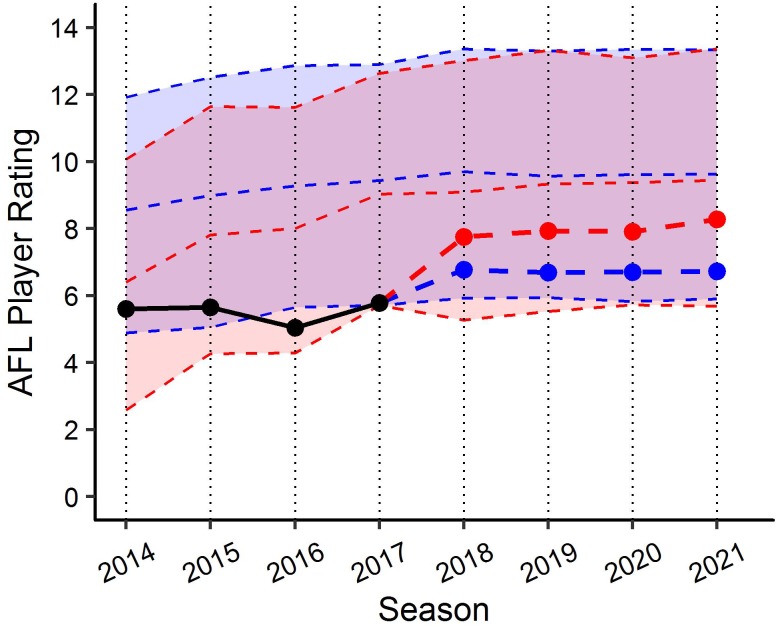
Benchmark levels of AFL Player Ratings for a specific player using the both the age (blue) and experience (red) linear mixed models. Black line represents actual performance to 2017. Blue and red points indicate expectation of performance from 2018 using each the age and experience models, respectively. Similarly, each ribbon represents fixed effects estimates based on characteristics of same player in each model.

By applying the PSRE and the fixed effect estimates from the linear mixed models, various applications can be created to benchmark player performance. For example, Figure 6 visualises the actual past performance and future player specific expectation of performance (fit and 90% PI) for a specific player, as compared to their fixed effect estimate of performance using model (1). This application indicates the player’s performance has been below the benchmark level of performance since 2014, but within the 90% PI, and is expected to remain fairly consistent in the three forecasted seasons. Figure 7 outlines how model (1) could be used for player comparison, indicating that the player in blue is likely to perform better in each of the forecasted seasons. Further, Figure 8 visualises the actual past performance and future player specific expectation of performance (fit only) for a specific player, using both the models based on age (blue) and experience (red).

Additionally, the PSRE provide a measure of player ranking, which adjusts for the individual fixed effects characteristics. Table 3 outlines the top five players in each positional roles, as determined by the average of the PSRE across the two linear mixed models. Player positional role was determined by the category in which they were categorised the most frequently over the five seasons.

**Table 3:** Top five players in each positional role, as determined by the average of the player specific random effects (PSRE) in each of the linear mixed models.

Player	Model 1 PSRE	Model 2 PSRE	Player	Model 1 PSRE	Model 2 PSRE
**General defender**			**General forward**		
	
Zac Williams	4.09	3.14	Brent Harvey	6.18	4.74
Adam Saad	3.30	3.22	Chad Wingard	4.31	3.26
Shaun Burgoyne	3.68	2.84	Eddie Betts	4.19	3.19
Brandon White	3.04	2.57	Luke Breust	4.32	3.02
Daniel Rich	2.97	2.57	Cyril Rioli	3.43	3.00
	
**Key defender**			**Key forward**		
	
Jeremy McGovern	4.44	4.11	Lance Franklin	5.21	4.51
Alex Rance	3.59	2.99	Jarryd Roughead	4.23	3.66
Tom McDonald	2.87	2.16	Justin Westhoff	4.22	3.10
Harris Andrews	3.04	1.87	Josh J. Kennedy	3.73	3.02
Josh Gibson	2.94	1.51	Jack Gunston	3.68	2.99
	
**Midfielder**			**Midfield-forward**		
	
Gary Ablett	8.29	6.96	Robbie Gray	4.75	3.76
Patrick Dangerfield	6.96	6.30	Dayne Zorko	3.71	3.88
Nat Fyfe	6.61	5.77	Sam Menegola	3.30	4.11
Scott Pendlebury	6.09	5.60	Christian Petracca	3.53	3.42
Marcus Bontempelli	5.44	4.49	Luke Dahlhaus	3.82	2.72
	
**Ruck**					
	
Todd Goldstein	4.70	3.68			
Nic Naitanui	4.29	3.57			
Sam Jacobs	3.60	2.19			
Aaron Sandilands	3.95	1.83			
Shane Mumford	3.52	1.94			

*Player positional role determined by the category in which they were categorised the most frequently over the five seasons.*

## Discussion

The primary aim of this study was to develop a model to objectively benchmark player performance whilst considering their age, experience, positional role, and both draft type and round in which they were selected. It also aimed to identify the stage of peak performance and specific breakpoints in player performance longitudinally. Separate linear mixed model analyses were implemented to benchmark performance based on the multifactorial fixed effects estimates. Segmented models were fit to these fixed effect estimates to determine if and where a change in the linear trend of performance progression occurs.

Visual inspection of the descriptive statistics in Figure 1A,B indicate that performance continues to improve throughout an AFL players career (as indicated by the gradual increase in average AFL Player Ratings for both age and experience, respectively). However, it must be noted that this type of analysis is susceptible to selection biases (Brander et al., 2014). Specifically, previous research has identified that these biases can be bought upon as a result of better-performing players typically having longer careers than other players (Bradbury, 2009; Dendir, 2016). Figure 3, 4 highlight this bias on the basis that player selection is a subjective identification of each clubs best performers. Specifically, Figure 3 outlines the proportion of players in the dataset, and indicates that there are less players across the sample in older and more experienced categories, respectively; however, Figure 4 shows that these older and more experienced players on average play more games per season. The substantially smaller interquartile ranges and presence of outliers in Figure 4B, as opposed to Figure 4A, indicates that despite showing similar increasing trends between the two distributions, there is less variance in matches played per season with respect to experience. However, this is somewhat expected due to the compounding nature of matches played per season, to total career matches. Visual inspection of the descriptive statistics in Figure 2A,B also indicates that performance differences are seen between varying levels of both draft and position, respectively. These findings align with previous literature investigating longitudinal player performance, and supports the use of a mixed model approach to account for fixed and PSRE (Bradbury, 2009; Dendir, 2016).

Each of the two linear mixed models provide context when looking to benchmark player performance longitudinally in AF. In addition to identifying a universal benchmark trend of performance longitudinally, the models produced in this study allow player specific values to be obtained, by adjusting each of the fixed effects relative to the player’s characteristics in each model. These player specific benchmarks allow for both retrospective assessment of a players past performance against expected performance, as well as to forecast player performance relative to expected characteristics (assumptions must be made with regards to positional role and experience to forecast). Applications of these models have the potential to be beneficial in supporting the decision making processes within professional AF organisations. Decisions relating to player recruitment and contracting could be objectively informed by gaining an understanding of the past and future potential performance of players, which the club maybe looking to recruit, resign or remove from their current playing squad. Though the examples provided in this study feature 90% PI, clubs/organisations wanting to be more aggressive with their predictions regarding expected performance could adapt the current models to include lower PI. Figure 6 provides a specific example of how this can be visualised. It outlines an actual player’s past performance (2014–2017) and expected future performance (2018–2021), and compares this to the benchmark level of performance based on the characteristics for that player. Alternatively, Figure 7 outlines an actual player’s past performance (2014–2017) and expected future performance (2018–2021), and compares this to the expected future performance (2018–2021) of a player who is yet to be drafted.

Though the identified breakpoints found in each model differ marginally to the findings of the *post hoc* Tukey test, both analyses indicate that there is a distinct change in the trend of player performance occurring in each model, occurring at around the age 22, and experience level 41–60, respectively. Specifically, they indicate that this change in the trend represents a point of marginal gains within each of the model, such that once these levels are reached the benchmark level of player performance is expected to somewhat plateau. This indication of marginal performance gains beyond these respective levels could have useful implications for both player development and player recruiting/contracting within professional AF. For example, clubs may look to persist with selection of players who are yet to reach these points of marginal gains (as opposed to older/more experienced players of similar ability), knowing that match opportunities are potentially more detrimental to development of the younger/less experienced players. In regards to player recruiting and contracting, clubs could look to use these breakpoints as an indication of whether the performance of current players and/or potential recruits is likely to continue to improve, or whether their performance has reached a point of marginal gains. Though only one breakpoint was identified for each model in this study, clubs/organisations wanting to further explore the longitudinal performance trends could adapt the current methodology to identify whether multiple breakpoints exist.

Despite minor differences, both the models measured longitudinally on each age and experience might be used for different operational purposes based on the preferences of the organisation. For example, due to the reliance of match opportunity for the model based experience, applications of this model may be more suited to benchmark the performance of players who have experienced long-term injuries or are mature aged recruits. Conversely, for those who have had sufficient match opportunities, the models based on age may be more suitable due to the more progressive nature of age as an independent variable. Figure 8 visualises this difference in the models through benchmarking the expected performance of a specific older age, but lowly experienced individual, using both models.

In addition to providing benchmark levels of performance, the models produced in this study also provide an indication of the point at which peak performance occurs longitudinally. Specifically, the findings imply that on average players reach their peak around the age of 22, or 60 matches experience. In comparison to previous literature, this point at which the average player reaches their peak age is younger than what has been identified in other dynamic team sports such as soccer (Dendir, 2016). Though this peak is identified earlier, there was no substantial drop-off in performance noted in this study, indicating that that peak performance in AF may be better outlined by a peak range. There is no literature available to make these comparisons in relation to a player’s match experience.

The PSRE outlined in each of the mixed models could also be used to rank players across the 2013–2017 seasons. Specifically, this type of ranking would be more generalisable than other ranking measures that do not adjust for fixed effects such as those used in our model. Thus it allows comparisons to be made between players across different ages, levels of experience, positional roles and draft selections. Table 3 outlined the top five players in each positional role. The table indicated that despite accounting for position, the top three midfielders still exhibited higher PSRE than any other players. As an indication of the face validity for these random effects to be used to rank players, each of these three outlined individuals have won the AFL’s award for the fairest and best player for one of the five seasons included in the dataset (Gary Ablett in 2013, Nat Fyfe in 2015 and Patrick Dangerfield in 2016).

Some limitations of this study should also be noted. Though mixed model approaches have been supported in previous literature to account for the fixed and random effects associated with longitudinal player performance; there is also an inherent understanding that the decline in performance after peak is often underestimated as a result of athlete drop out. For example, only the most successful athletes continue to get renewed playing contracts, and are subsequently selected to play at the elite level. Thus meaning that there is likely some level of performance deterioration that goes unnoticed by the model beyond certain ages/levels of experience. Another limitation is that the methodology could include additional metrics, such as time on ground or spatiotemporal data, potentially allowing for further explanation of the results. Future work in dynamic team sports should focus on the continual development of improving objective player performance rating models, as well as decision support applications to assist with operational decision-making in professional sporting organisations. In AF specifically, the development of these objective player performance rating models could look to include further positioning dynamics, similar to that in other team sports (Gonçalves et al., 2017; Memmert et al., 2017).

## Conclusion

This study produced two types of models benchmarking player performance in the AFL. The first method utilised two separate linear mixed models to identify the effect of individual characteristics on player performance. Each of these models could be used to identify how a player’s performance compares to individualised benchmarks, or to forecast future potential performance. The second method utilised segmented models, finding a point of marginal gains within longitudinal performance of both age and experience. The implementation of these methodologies may provide valuable knowledge for professional AFL organisations. Implications of their use could assist with organisational decisions relating to player recruitment, contracting and development. Future work should focus on the refinement of the models produced in this study as additional seasons of data become available.

## Author Contributions

SM and SR conceived and designed the study. SM compiled the data, conducted the statistical analyses, and wrote the bulk of the manuscript. SR oversaw the data collection and statistical analyses, and contributed substantially to the writing of the manuscript. SK contributed significantly to the methodology, and assisted with writing of the “Materials and Methods” section.

## Conflict of Interest Statement

The authors declare that the research was conducted in the absence of any commercial or financial relationships that could be construed as a potential conflict of interest.

## APPENDICES

**Table A1:** Descriptions of the seven positional roles used in this study.

Positional roles	Description
General defender	Plays a role on opposition small-medium forwards and usually helps create play from the backline
Key defender	Plays on opposition key forwards with the primary role of nullifying his opponent
General forward	Plays predominantly in the forward half of the ground but with more freedom than a key forward
Key forward	Plays predominantly as a tall marking target in the forward line
Midfielder	Spends the majority of time playing on the ball or on the wing
Midfielder-forward	Splits time equally between the forward line and the midfield. Often lines up on the half-forward flank but plays a significant amount of time in the midfield
Ruck	Has the primary role of competing for hit-outs at a stoppage

**Table A2:** Descriptions of the three annual draft methods to enter an AFL list.

Draft type	Club participation	Trading of picks	Further description
National draft	Compulsory draft. Each club must exercise a minimum of three selections	Picks can be traded between clubs	Players selected by a club become ineligible to be included on the primary list of any other club for a period of two seasons. For the most part this draft consists of players finishing secondary school, who have been competing in elite junior feeder competitions
Preseason draft	Non-compulsory draft	Picks cannot be traded between clubs	Players selected by a club become ineligible to be included on the primary list of any other club for a period of two seasons. For the most part this draft consists of players who missed out on selection in the National Draft
Rookie draft	Non-compulsory draft	Picks cannot be traded between clubs	Players selected become part of the clubs rookie list, and cannot compete within the AFL until being promoted to the clubs primary list. For the most part this draft consists of players who missed out on selection in the National Draft or older players from second tier competitions

In all three drafts, clubs select players in the reverse order to which they finished on the final premiership ladder in the previous AFL season. To be eligible for selection, a player must be 18 years of age before the 31st of December following the national draft selection meeting.
